# The Hidden Face of Lyme Disease: Neuroinfection With Cranial Nerve Involvement

**DOI:** 10.7759/cureus.103747

**Published:** 2026-02-16

**Authors:** Michal Sobczak, Aleksandra Morajko, Nina Urantowka, Karolina Moszko, Beata Labuz-Roszak

**Affiliations:** 1 Faculty of Medicine, University of Opole, Opole, POL; 2 Department of Neurology, St. Jadwiga Regional Specialized Hospital, Institute of Medicine, University of Opole, Opole, POL

**Keywords:** cranial nerve involvement, cranial nerve vi palsy, lyme disease, lyme neuroborreliosis, neurology

## Abstract

The article presents the case of a 54-year-old woman who presented to the hospital for a diagnosis of persistent headaches. The patient had been unsuccessfully treated for two months, developing further complications in the form of visual disturbances and abducens nerve palsy. The patient reported a recent tick bite. Imaging studies in the form of a magnetic resonance imaging (MRI) of the brain, magnetic resonance angiography (MRA), and transcranial Doppler ultrasound showed no abnormalities. In the next step, a lumbar puncture was performed, which yielded clear cerebrospinal fluid (CSF) with marked lymphocytic pleocytosis (122 cells/μL). CSF analysis confirmed the intrathecal synthesis of antibodies to *Borrelia*. Based on the clinical picture including the complications the patient developed, along with a history of tick bites, a diagnosis of neuroborreliosis with cranial nerve involvement was made. After starting antibiotic therapy with ceftriaxone, the patient showed significant clinical improvement after a few days. It should be remembered that in the course of Lyme disease, the nervous system can often be involved, resulting in many bothersome and unusual symptoms, which often make it difficult to make a correct diagnosis.

## Introduction

Lyme disease is an infectious disease caused by different types of *Borrelia burgdorferi* spirochetes. They are transmitted to humans through the bite of a spirochete-infected tick [1]. Lyme disease is characterized by a variable course of symptom accumulation, which most often begins with a single skin lesion in the form of erythema migrans [2]. In the situation of invasion of the central and/or peripheral nervous system by the spirochetes, neurological symptoms will develop, which may be the only symptoms of the disease [3]. The course of neuroborreliosis can be nonspecific; hence, there are often differences in the clinical course of the disease. In adults, neuroborreliosis most often manifests as nerve root inflammation or limb paresis. In children, on the other hand, the most common course is meningitis with associated or isolated facial nerve inflammation [4]. A confident diagnosis of active neuroborreliosis should be based on the two-step finding of specific antibodies to *Borrelia burgdorferi* in the cerebrospinal fluid (CSF) using enzyme-linked immunosorbent assay (ELISA) and Western blot serological tests [5]. Lumbar puncture is also used in the diagnosis, where pleocytosis in the cerebrospinal fluid will be characteristic of neuroborreliosis [6]. The treatment of neuroborreliosis is based primarily on antibiotic therapy, most often with ceftriaxone administered intravenously. Clinical studies have shown that oral doxycycline, a tetracycline antibiotic, is equally effective [7]. This case report highlights the story of a patient with neuroborreliosis presenting with unusual clinical manifestations as a result of a tick bite in the past. Initial clinical manifestations, including nystagmus and abducens nerve palsy, failed to suggest an association with a prior *Borrelia burgdorferi*-infected tick bite and had the potential to lead to diagnostic misinterpretation.

## Case presentation

A 54-year-old woman was admitted to our department for the evaluation and management of persistent headaches. Her medical history revealed ongoing, treatment-resistant headaches persisting for approximately two months, during which she also developed visual disturbances, specifically diplopia. She additionally reported a recent tick bite. Outpatient serological testing demonstrated positive immunoglobulin G (IgG) antibodies and negative IgM antibodies against *Borrelia burgdorferi*, confirmed by both enzyme-linked immunosorbent assay (ELISA) and Western blot methods. Her past medical history was notable for glaucoma.

**Figure 1:**
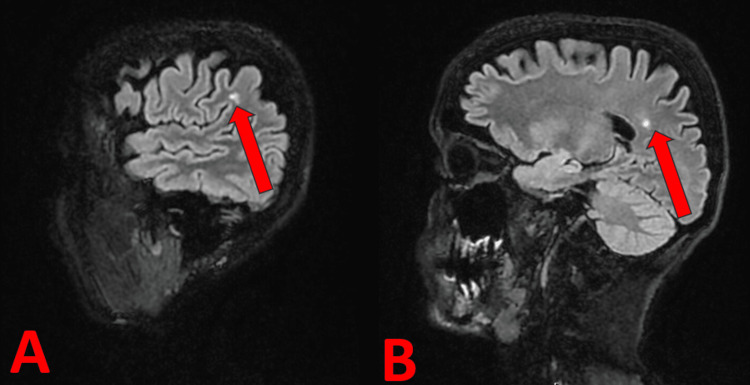
MRI (A) The MRI scan of the patient's brain (frontal section). The red arrow indicates small isolated hyperintense areas in the white matter. The scans come from the hospital records. (B) The MRI scan of the patient's brain (sagittal section). The red arrow indicates small isolated hyperintense areas in the white matter MRI: magnetic resonance imaging

Magnetic resonance imaging (MRI) of the brain (Figure 1) revealed three small isolated areas of hyperintensity in the deep white matter of the right cerebral hemisphere, likely of vascular origin and considered age-appropriate. No other structural abnormalities were identified. Contrast-enhanced imaging showed normal cerebral vasculature, and cerebrospinal fluid spaces appeared within normal limits.

**Figure 2:**
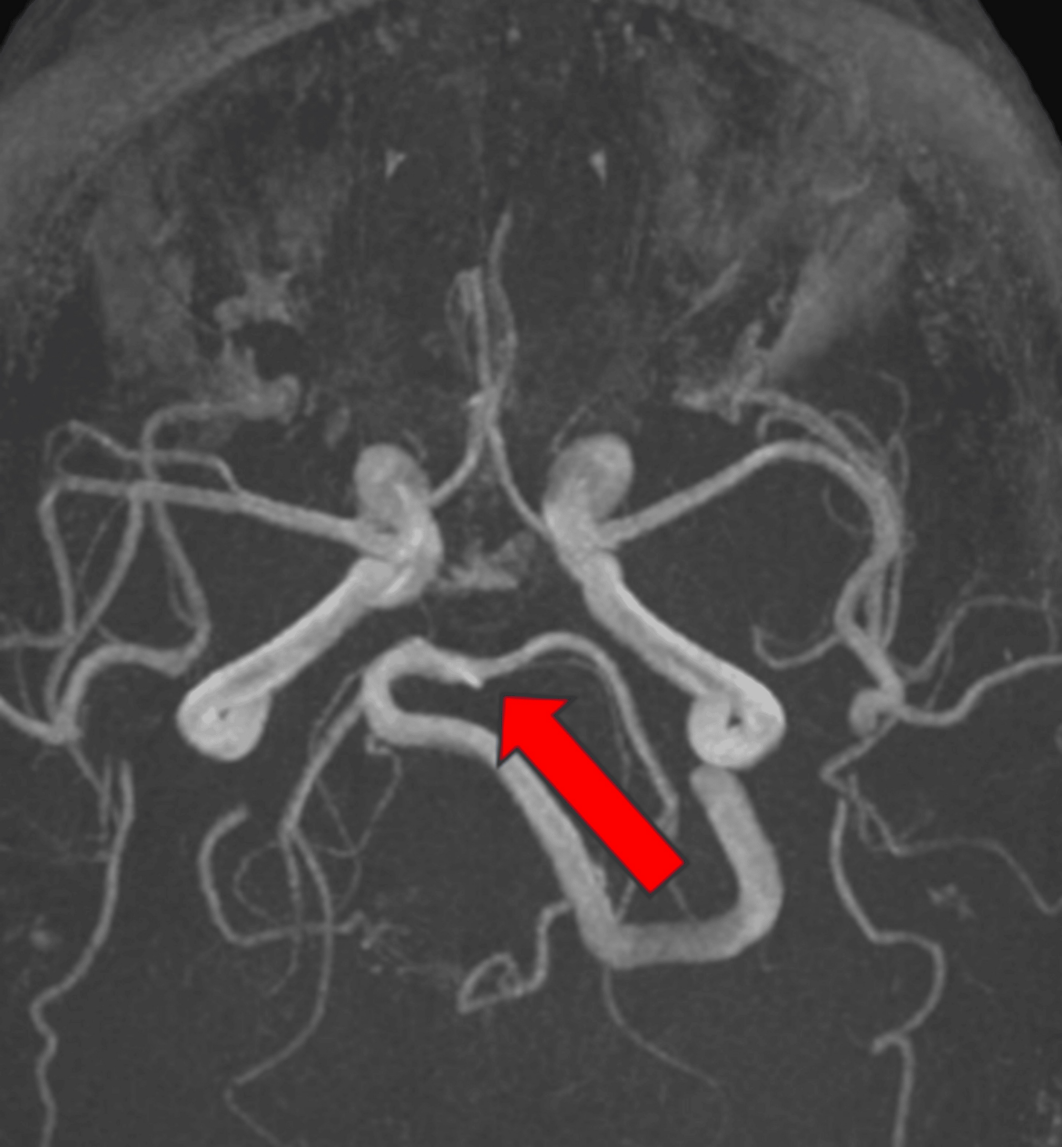
Magnetic resonance angiography (MRA), axial section Hypoplasia of the right vertebral artery (VA), with a narrowed lumen measuring approximately 1 mm in the V3 segment and faint, threadlike contrast uptake in the V4 segment

Magnetic resonance angiography (MRA) of the cerebral vessels (Figure 2) demonstrated hypoplasia of the right vertebral artery (VA), with a narrowed lumen measuring approximately 1 mm in the V3 segment and faint, threadlike contrast uptake in the V4 segment. This was interpreted as a developmental variant. The remaining arterial and venous structures were unremarkable.

Transcranial Doppler ultrasound confirmed normal, symmetrical flow in the common, internal, and external carotid arteries. A discrepancy in vertebral artery size was observed, with the right VA measuring 1.6 mm and the left VA measuring 4.5 mm, both following normal anatomical courses. Doppler analysis revealed standard flow velocities in the anterior (ACA), middle (MCA), and posterior cerebral arteries (PCA); however, the right VA exhibited elevated resistance and pulsatility indices.

**Figure 3:**
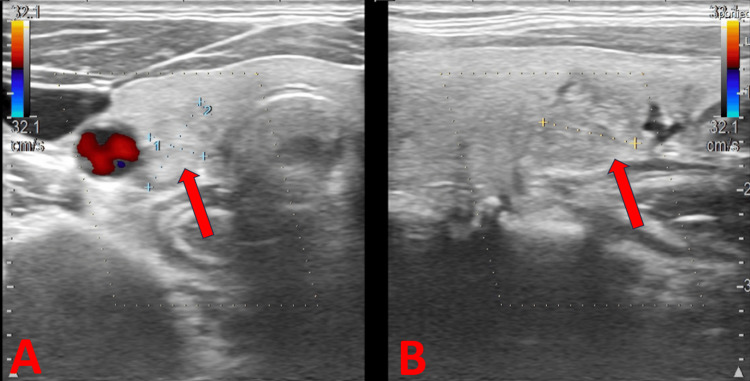
UDP (A) Focal change in the thyroid gland, transverse section. (B) Focal change in the thyroid gland, longitudinal section UDP: Doppler ultrasound

A thyroid ultrasound (Figure 3) incidentally revealed a focal lesion with irregular borders in the posterior part of the right lobe (6 × 10 × 10 mm), necessitating further evaluation by an endocrinologist.

A lumbar puncture was performed, yielding clear cerebrospinal fluid (CSF) with marked lymphocytic pleocytosis (122 cells/μL; normal range: 0-5 cells/μL). CSF analysis confirmed the intrathecal synthesis of anti-*Borrelia* antibodies, supporting the diagnosis. Western blot analysis was performed to detect pathogen-specific immunoglobulins (IgG and IgM) directed against *Borrelia burgdorferi* in peripheral blood samples. The assay served as a confirmatory method for previously obtained enzyme-linked immunosorbent assay (ELISA) results. Pathogen-derived antigens were separated by electrophoresis, transferred onto a membrane, and subsequently incubated with patient sera. Immunoreactive antigen-antibody complexes were visualized using appropriate secondary antibodies. The method is characterized by high analytical specificity and methodological precision.

The patient was initiated on targeted antibiotic therapy with intravenous ceftriaxone (Biotrakson). After six days of treatment, she demonstrated significant clinical improvement and was discharged in good general condition, with the remainder of the antibiotic therapy to be completed at home, in accordance with the guidelines.

Taking into account the clinical presentation, including persistent headaches, diplopia, and abducens nerve palsy, along with her tick bite history, serological findings, CSF analysis, and imaging results, a diagnosis of neuroborreliosis with cranial nerve involvement was established.

## Discussion

Lyme borreliosis is an illness caused by the spirochete *Borrelia burgdorferi*, with ticks of the *Ixodes* genus serving as its vector. The disease affects multiple organ systems, including the musculoskeletal system, skin, heart, nervous system, and visual system. Lyme disease occurs in regions where ticks are present, namely, North America, Europe, and Asia [8].

Lyme neuroborreliosis (LNB) may present in both early and late stages of infection, and it can also be the first manifestation of the disease [9]. Nearly half of the patients associate their symptoms with a previous tick bite, while 20%-30% report cutaneous manifestations, such as erythema migrans, corresponding to stage I of infection. The majority of the patients present with early Lyme neuroborreliosis (stage II), defined by symptoms lasting less than six months, whereas late-stage Lyme neuroborreliosis (stage III), with a duration of six months to several years, occurs in fewer than 5% of cases [10].

The most common symptoms include cranial neuropathy of the facial nerve (41%-68%), radiculopathy (30%-66%), isolated meningitis (6%-10%), and encephalitis (<1%-12%) [5]. In the late phase, encephalomyelitis may develop, accompanied by spastic symptoms, gait disturbances, and bladder dysfunction. Occasionally, pain in various locations and isolated meningitis may also occur [11].

Meningitis and radiculopathy with severe radicular pain and cranial nerve palsy are described as Garin-Bujadoux-Bannwarth syndrome, which represents the most common neurological presentation in adults following erythema migrans. A relatively rare but often overlooked aspect of neuroborreliosis includes ocular manifestations such as blurred vision, diplopia, abducens nerve palsy, or strabismus [11]. Cranial nerve involvement is observed in approximately 60% of the patients and may, in some cases, constitute the sole clinical manifestation. Among affected nerves, the facial nerve is predominantly compromised, often bilaterally, while the involvement of other cranial nerves occurs considerably less frequently [3]. In children, neuroborreliosis is most often diagnosed during its early phase. The most characteristic symptoms in pediatric patients include facial nerve palsy accompanied by meningitis; however, weakness, mood swings, and loss of appetite may also occur [12]. Due to the nonspecific nature of many symptoms, such as headache, fatigue, and cognitive impairment, which are also common in other conditions, diagnosing LNB can be challenging [13].

The basis of laboratory diagnosis for *Borrelia burgdorferi* infection is a two-tiered antibody detection system. The first step involves an enzyme immunoassay (EIA), followed by confirmation using the Western blot method. The sensitivity of this two-step testing is low in early-stage disease, meaning that a negative result does not rule out infection. However, sensitivity increases as the disease progresses [14]. IgM antibodies to *Borrelia burgdorferi* can typically be detected from the third week after the tick bite, while IgG antibodies become detectable from around the sixth week, with higher concentrations typical of later-stage manifestations. In patients with persistent LNB symptoms, the presence of IgG antibodies is considered diagnostic. Conversely, isolated elevated IgM antibody titers may indicate early infection but are not reliable for diagnosing persistent or late-stage disease [15].

Cerebrospinal fluid (CSF) analysis is a key diagnostic tool in neuroborreliosis. Typical findings include pleocytosis, with leukocyte counts ranging from 10 to 1000 cells/mm³ (with lymphocytic predominance), as well as elevated protein levels [16].

According to these guidelines, LNB can be diagnosed when the following three criteria are met: the presence of neurological symptoms consistent with neuroborreliosis (after excluding other causes), pleocytosis in the cerebrospinal fluid, and the presence of *Borrelia burgdorferi*-specific antibodies in the CSF (produced intrathecally).

Meeting all three criteria allows for a definite diagnosis of neuroborreliosis. If only two out of the three criteria are met, a possible neuroborreliosis is considered; however, the test for *Borrelia burgdorferi*-specific antibodies should then be repeated after six weeks.

Imaging studies are also helpful in diagnosing LNB, as they may reveal central nervous system changes or cerebrovascular involvement. Magnetic resonance imaging (MRI) plays an important role both in the diagnostic process and in monitoring disease progression. Given the wide range of clinical symptoms and laboratory parameters, the European Federation of Neurological Societies (EFNS) published diagnostic guidelines for LNB in 2010 [10].

The cornerstone of LNB treatment is antibiotic therapy, which is typically initiated in patients presenting with neurological symptoms consistent with *Borrelia burgdorferi* infection, accompanied by inflammatory changes in the CSF and positive serological test results. If neuroborreliosis is suspected, a thorough differential diagnosis should be conducted before initiating antibiotic treatment to exclude other potential causes of the symptoms [17]. Most studies recommend a treatment duration of 10-14 days, although in some cases it may be extended up to 28 days. Commonly used antibiotics include oral doxycycline and intravenous ceftriaxone, cefotaxime, or penicillin G. In early-stage LNB, 90% of the patients experience symptom resolution within one year following treatment. However, late-stage neuroborreliosis may leave residual neurological deficits even a year after therapy [18]. These are typically nonspecific symptoms such as fatigue and cognitive dysfunction, referred to as post-treatment Lyme disease symptoms (PTLDS) or post-treatment Lyme disease syndrome (PTLDS) [19]. Despite previous antibiotic treatment, there is currently no established therapeutic protocol for managing PTLDS, and many patients do not receive a definitive diagnosis or effective care, which can significantly impair the quality of life and daily functioning [20].

## Conclusions

Lyme neuroborreliosis is a complex condition with diverse clinical presentations, requiring careful clinical evaluation and diagnostic confirmation. The disease manifests differently in various age groups, and its complications may affect multiple organs. The diagnosis of neuroborreliosis is based both on the neurological symptoms presented by the patient and on the presence of pleocytosis in the cerebrospinal fluid, as well as the presence of *Borrelia burgdorferi*-specific antibodies in the CSF. Early detection and appropriate antibiotic therapy significantly improve outcomes, particularly in early-stage disease. Through differential diagnostic evaluation, we excluded the presence of focal lesions involving the skull base or brainstem that could mimic similar clinical manifestations. However, ongoing research is needed to better understand and manage post-treatment symptoms, especially in patients affected by post-treatment Lyme disease syndrome (PTLDS). In light of the increasing incidence of these diseases, there is an urgent need for further systematic research to comprehensively characterize their adverse effects and to accurately assess both short- and long-term health consequences, as well as the impact of early diagnosis.
